# Evaluation of Microcirculation in Optic Nerve Head Using Laser Speckle Flowgraphy in Active Thyroid Eye Disease

**DOI:** 10.1155/2022/9115270

**Published:** 2022-03-16

**Authors:** Margaret Ming-Chih Ho, Yueh-Ju Tsai, Yen-Chang Chu, Yi-Lin Liao

**Affiliations:** ^1^Department of Ophthalmology, Chang Gung Memorial Hospital, Linkou, Taiwan; ^2^College of Medicine, Chang Gung University, Taoyuan, Taiwan

## Abstract

**Background:**

Laser speckle flowgraphy (LSFG) can be used to estimate optic nerve blood flow. This study used LSFG to evaluate optic nerve microcirculation in patients with thyroid eye disease (TED).

**Methods:**

This was a retrospective review of patients with active TED who underwent LSFG between October 2020 and June 2021. The mean blur rate (MBR) for different severities of active TED was analyzed by one-way analysis of variance (ANOVA).

**Results:**

A total of 30 patients (60 eyes) with a diagnosis of active TED who underwent LSFG were included. The mean age was 49 (range, 33–74) years. Mean best-corrected visual acuity was the worst in the group with sight-threatening active TED (0.29 ± 0.33 logarithm of the minimum angle of resolution, *p* = 0.01). The MBR-overall was the highest in the group with mild active TED (28.5 ± 2.7), followed by that in the moderate to severe (23.6 ± 3.2), and in the sight-threatening (20.2 ± 4.3) active TED groups (*p* < 0.001). The MBR-vessel was 57.1, 47.0, and 39.3 in the mild, moderate to severe, and sight-threatening active TED groups, respectively (*p* < 0.001). The MBR-tissue was 16.9, 14.4, and 12.0 in the mild, moderate to severe, and sight-threatening active TED groups, respectively (*p* < 0.001).

**Conclusions:**

This study demonstrates that optic nerve blood flow is lower with more severe active TED. In addition, LSFG is an effective, objective, and noninvasive method for evaluating the severity of TED.

## 1. Introduction

Thyroid eye disease (TED) is an autoimmune inflammatory disease characterized by proptosis, lid retraction, and diplopia (1). TED occurs in 35% (2) of patients with Graves' disease and may affect the patient's appearance, causing ocular discomfort and leading to dysthyroid optic neuropathy (DON). DON, a severe complication of TED, has been demonstrated to occur in 4–8% (3–5) of the population with TED and may lead to a decrease in color vision, visual field defect, and even visual loss without early and prompt management (5, 6).

Currently, the diagnosis of TED is generally based on clinical presentation. The European Group on Graves' Orbitopathy (EUGOGO) classified TED into mild, moderate to severe, and sight-threatening according to the degree of lid retraction, soft tissue involvement, proptosis, and diplopia (7). Visual field changes, relative afferent pupillary defects, diminished color vision, and radiological hypertrophy of the extraocular muscle might also indicate progression of the disease (6, 8). However, as far as we are aware of, none of the aforementioned parameters can be used to objectively quantify the severity of the disease individually. Color Doppler ultrasound has been used to measure orbital blood flow changes in patients with TED, but the relationship between orbital blood flow and the severity of TED remains unclear (9–11).

Laser speckle flowgraphy (LSFG) is based on the laser speckle phenomenon and is a noninvasive instrument that can measure two-dimensional relative blood flow velocity of the ocular microcirculation in real time (12, 13). The mean blur rate (MBR), which represents the velocity of optic nerve blood flow, has been previously applied to monitor the severity of glaucoma (14, 15), evaluate ischemic optic neuropathy (16, 17), and detect retinal diseases (18). Although LSFG can be used to estimate optic nerve blood flow, to the best of our knowledge, it has never been used to monitor blood flow changes in patients with TED or to evaluate the severity of TED.

The aim of this study was to evaluate optic nerve microcirculation through LSFG in patients with active TED of differing degrees of severity. We hypothesized that patients with more severe active TED would have lower MBR, which could be determined using LSFG.

## 2. Materials and Methods

### 2.1. Characteristics of Study Patients

This was a retrospective study of patients with active TED who underwent LSFG. Electronic medical charts for patients between October 2020 and June 2021 at Linkou Chang Gung Memorial Hospital, the largest tertiary referral hospital in Taiwan, were reviewed. The study design conformed to the principles of the Declaration of Helsinki and was approved by the Chang Gung Medical Foundation's Hospital Ethics Committee on Human Research (IRB number: 202101159B0).

We recruited patients with active TED. Active TED was defined as an inflammatory score ≥ 4 on the VISA grading system (19). The VISA inflammatory score organizes the clinical features of TED into 4 discrete groupings: V (vision, dysthyroid optic neuropathy), I (inflammation, congestion), S (strabismus, motility restriction), and A (appearance, exposure). Patients with a history of glaucoma, ocular hypertension, ocular surface disease, or optic nerve diseases other than DON were excluded. Patients who could not undergo LSFG were also excluded.

Demographic data and information on previous systemic and ocular diseases, steroid use, surgical treatment for Graves ophthalmopathy, clinical presentations, and comprehensive ocular examination results comprising best-corrected visual acuity (BCVA), intraocular pressure, Hertel exophthalmometry, the Ishihara color test, and the diplopia test were obtained from participants' medical records. BCVA was measured using the Snellen chart. Mean arterial pressure (MAP) was calculated as DBP + 1/3(SBP–DBP), and mean ocular perfusion pressure (MOPP) was calculated as 2/3[DBP + 1/3 (SBP − DBP) − IOP] (20, 21).

### 2.2. EUGOGO Classification

Patients with active TED were divided into groups by EUGOGO classification (7). The EUGOGO classifies TED severity as follows:

1. TED is considered mild if the patient has one or more of the following

- Minor lid retraction (<2 mm)
- Mild soft-tissue involvement
- Exophthalmos <3 mm above normal for race and gender
- No or intermittent diplopia with corneal exposure responsive to lubricants

(2) TED is considered moderate to severe if the patient has two or more of the following

- Lid retraction of ≥2 mm
- Moderate or severe soft-tissue involvement
- Exophthalmos ≥3 mm above normal for race and gender
- Intermittent or constant diplopia

(3) TED is considered sight-threatening if the patient has previously experienced DON and/or corneal breakdown

### 2.3. LSFG and Clinical Settings

**Figure 1:**
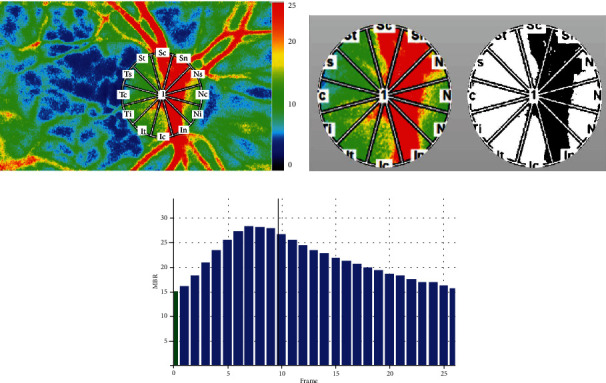
Analysis of pulse waveforms for the optic nerve head (ONH) using laser speckle flowgraphy (LSFG). (a) A representative color-coded composite map was used to record the mean blur rate (MBR). Red represents high MBR, and blue represents low MBR. (b) MBR was calculated by placing a rubber band around the ONH. The segmentation of the vessel area (black area) and tissue area (white area) of the ONH is shown in binary format. (c) A pulse waveform of MBR for one cardiac cycle.

All the patients in our study underwent LSFG. The instrument was equipped with a fundus camera with a diode laser (wavelength, 830 *μ*m) as the light source and a charge-coupled sensor (750 pixels wide × 360 pixels high) as the detector. A speckle pattern is produced by the laser, whose interference is scattered by the movements of erythrocytes; the interference pattern can be detected by the detector. MBR, acquired at 30 frames/second over a 4 second period, represents the velocity of optic nerve head (ONH) blood flow (Figure 1). MBR can be expressed as overall MBR (MBR-overall) or divided into that of the vessel areas (MBR-vessel) or tissue areas (MBR-tissue).

In our study, the patients' heart rate, systolic blood pressure, and diastolic blood pressure were measured after the patients had rested in a quiet room for 10 minutes before LSFG. MBR values were measured with the LSFG RetFlow (NIDEK Co., Ltd. Gamagori, Aichi, Japan). Both eyes of the patients were measured. An average of three successive measurements in each eye was required to determine MBR values.

### 2.4. Statistical Analyses

Descriptive statistics were used to evaluate patient demographics and baseline clinical examination results at the time of presentation. The intraocular pressure in upward gaze, MBR-overall, MBR-vessel, and MBR-tissue following normal distributions was analyzed through one-way analysis of variance (ANOVA). Because the patients' BCVA, age, and intraocular pressure in primary gaze did not follow a normal distribution, the Kruskal–Wallis test was used to evaluate differences among the active TED severity groups. Independent *t* tests or Mann–Whitney *U* tests were used to evaluate the differences between any two active TED severity groups. A chi-squared test was used to evaluate sex differences among TED severity groups. The significance level was set at *p* = 0.05. Statistical analyses were performed using MedCalc Statistical Software version 20.009 (MedCalc Software Ltd., Ostend, 2021).

## 3. Results

A total of 54 patients with a diagnosis of TED who underwent LSFG were included. However, 19 were excluded because of inactive TED (VISA inflammatory score < 4), and five were excluded because of incomplete medical records. Therefore, 30 patients (60 eyes: 11 male and 19 female) were included. The mean age was 49 years (range, 33–74 years).

### 3.1. Clinical Characteristics in Different TED Severity Groups

The clinical characteristics and MBRs of the different TED severity groups are listed in Table 1. Mild, moderate to severe, and sight-threatening TED accounted for 12 (20%), 31 (52%), and 17 (28%) eyes, respectively. The mean age of the sight-threatening TED group was 57.3 years, higher than both the moderate to severe (46.8 years) and the mild (42.6 years) TED groups (*p* = 0.003). Differences in the prevalence of smoking among the different groups were not observed (*p* > 0.999). Of the patients who received high-dose intravenous steroid treatment, 12 (70.6%) were in the sight-threatening TED group, 15 (48.4%) in the moderate to severe TED group, and 3 (25%) were in the mild TED group. Five patients (29.4%) in the sight-threatening group had a history of orbital decompression, whereas one patient (3.2%) in the moderate to severe group had such a history.

Mean systolic blood pressure in the three groups was 98.5 ± 34.6, 140.4 ± 24.4, and 122.3 ± 22.8 mmHg in the mild, moderate to severe, and sight-threatening groups, respectively (*p* = 0.002); mean diastolic blood pressure was 86.2 ± 20.9, 82.3 ± 15.1, and 74.0 ± 10.5 mmHg in the mild, moderate to severe, and sight-threatening groups, respectively (*p* = 0.503). MAP was 85.5 ± 10.3, 100.7 ± 15.3, and 90.1 ± 12.3 mmHg in the mild, moderate to severe, and sight-threatening groups, respectively (*p* = 0.119). MOPP was 59.8 ± 34.2, 49.9 ± 10.5, and 40.4 ± 8.7 mmHg the in mild, moderate to severe, and sight-threatening groups, respectively (*p* = 0.364). The mean heart rate was 68.8 ± 14.6, 69.8 ± 9.2, and 62.5 ± 11.6 beats per minute in the mild, moderate to severe, and sight-threatening groups, respectively (*p* = 0.890). The correlation coefficients between clinical characteristics and MBRs are listed in Supplemental Table 1.

### 3.2. BCVA, Intraocular Pressure, and MBR in the TED Severity Groups

**Figure 2:**
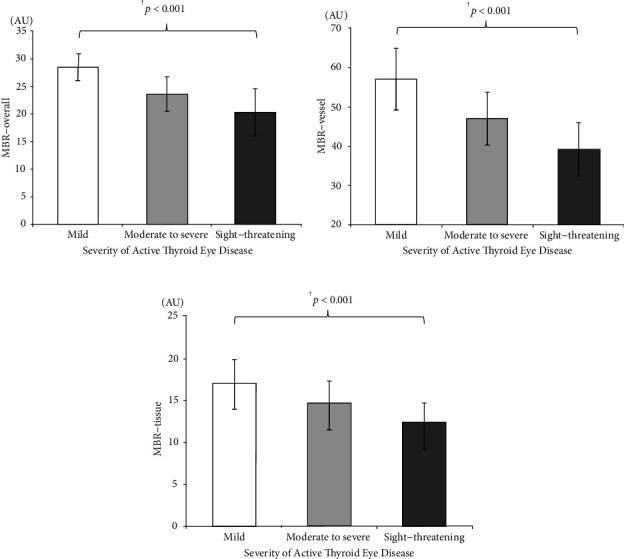
Mean blur rate (MBR) and different severities of active thyroid eye disease (TED). MBR-overall (a), MBR-vessel (b), and MBR-tissue (c) were significantly lower with more severe active TED.

The mean BCVA was the lowest in the sight-threatening group (logMAR 0.29 ± 0.33, *p* = 0.01). The sight-threatening group had the highest mean intraocular pressure in both primary (19.5 ± 3.4 mmHg, *p* = 0.03) and upward gaze (25.7 ± 4.9 mmHg, *p* = 0.03). The MBR-overall was the highest in the mild TED group (28.5 ± 2.7), followed by the moderate to severe (23.6 ± 3.2) and sight-threatening (20.2 ± 4.3) groups (*p* < 0.001; Figure 2(a)). The MBR-vessel was 57.1, 47.0, and 39.3 in the mild, moderate to severe, and sight-threatening groups, respectively (*p* < 0.001; Figure 2(b)), and the MBR-tissue was 16.9, 14.4, and 12.0 in the mild, moderate to severe, and sight-threatening groups, respectively (*p* < 0.001; Figure 2(c)). The statistical difference between each two groups is listed in Supplemental Figure S1. BCVA, intraocular pressure, and MBR values in the three active TED groups are listed in Table 1. The correlation coefficients between the ocular findings and the MBRs are listed in Supplemental Table 1.

## 4. Discussion

This study reviewed the ONH blood flow in patients with active TED who underwent LSFG measurements. To the best of our knowledge, this is the first study to objectively quantify ONH blood flow using LSFG in TED and statistically identify ocular blood flow changes in different severities of active TED. Using ANOVA, we found that MBR-overall, MBR-vessel, and MBR-tissue were lower in eyes with more severe active TED. The MBR can quantitatively measure blood flow velocity of the ONH in LSFG examinations (14, 15). Lower MBRs may indicate slower ONH blood flow velocities in patients with more severe active TED. Our results support that patient with more severe TED suffered from more ischemic status of optic nerve microcirculation.

Changes in orbital blood flow in patients with TED have been reported in previous studies (9–11, 22–24). Using color Doppler sonography, Somer et al. (23) reported lower mean superior ophthalmic vein blood flow velocity in patients with TED compared with those without TED. Konuk et al. (9) observed a significantly lower superior ophthalmic vein blood flow velocity in patients with sight-threatening TED than in those without sight-threatening TED. Lešin et al. (10) reported a decrease in the flow velocity of the ophthalmic artery and central retinal artery in patients with early sight-threatening TED, which suggested that changes in the orbital vasculature might be the first sign of sight-threatening TED. The decrease in MOPP resulted from a combination of one or more contributing factors, including the inflammatory process, passive compression by enlargement of the extraocular muscles, and increased orbital pressure (25). Using LSFG, we found a prominent trend of lower ocular blood flow velocity at more severity levels of active TED, supporting the theory of vascular insufficiency during the progression of active TED.

MAP is defined as the average arterial pressure throughout one cardiac cycle and can be affected by cardiac output and systemic vascular resistance (20). MOPP is a calculated value that is affected by MAP and IOP. A decrease in MOPP was indicated as a risk factor for the prevalence and progression of glaucoma because such a change may indicate ONH blood flow dysregulation in glaucoma patients (26). However, in patients with active TED, MOPP may be affected by multiple factors, including orbital inflammation, passive compression by enlargement of the extraocular muscles, and increased orbital pressure. MOPP is not an accurate indicator of ocular microcirculation because it is determined solely on the basis of blood pressure and intraocular pressure readings. Nevertheless, using the LSFG examination, we were able to measure the ocular microcirculation quantitatively and objectively, and this may help evaluate patients with active TED.

In comparison with color Doppler imaging (9, 10, 23), the advantages of LSFG are the high reproducibility and objectivity of measurements (27, 28). Furthermore, the penetrating long-wavelength laser used in LSFG may accurately examine the ONH without effects caused by the configuration of the disc. Moreover, LSFG may permit the evaluation of the overall ONH microcirculation rather than that of a single artery or vein, which may help reduce measurement error. A previous study used optical coherence tomography angiography to evaluate choroidal thickness, which can reflect choroidal vasculature changes in active TED (29). However, adequate visualization of the entire choroid is necessary for accurate measurements (30). Because LSFG measures perfusion of only the ONH, the visualization of the choroid is not needed.

In previous studies, age and sex have been considered as factors affecting LSFG results (31, 32). Iwase et al. (32) reported that women had a higher MBR-overall than men. Aizawa et al. (31) found that age was correlated with MBR-vessel and that sex was correlated with both MBR-overall and MBR-vessel. They also discovered that MBR-tissue was independent of both age and sex. In our study, age and TED severity were significantly correlated because of the clinical nature of TED (33). The age differences among groups might partially affect their MBR-overall and MBR-vessel results. However, the MBR-tissue, which is statistically independent of age, was also significantly lower for more severe TED. To further reduce the effects of age variability on our study, we conducted a small subanalysis by matching patients by age, with the difference in matched ages in the subanalysis group being not more or less than 2 years (Supplemental Table 2). In this small subanalysis, the MBR-overall, MBR-vessel, and MBR-tissue were all significantly lower in the severe active TED group. This result demonstrates that the decreases in MBR observed in our study were mainly the result of the severity of the disease rather than the effect of the ages of the patients in each group. Our study did not find a significant correlation between sex and TED severity.

The limitation of this study includes its retrospective nature. Moreover, because this study was conducted at a referral hospital, patients with mild active TED tended to be followed up at local hospitals, which limited the number of cases in the mild TED group. Besides, axial length was not controlled because this was a retrospective study; axial length is not a routine examination for patients with active TED. This may lead to bias due to the potential effect of axial length on MBR. Nevertheless, to the best of our knowledge, this study was the first to demonstrate the relationship between ONH blood flow and the severity of active TED and to use LSFG to evaluate patients with TED. Further longitudinal studies are required to evaluate ONH blood flow changes during TED progression.

## 5. Conclusions

Active TED has variable clinical manifestations. Our study demonstrates that a decrease in ONH perfusion is correlated with more severe TED. Moreover, LSFG is an effective, objective, and noninvasive method for evaluating patients with TED. We believe that our results may help clinicians to evaluate the severity of active TED more easily and accurately.

## Figures and Tables

**Table 1:** Clinical characteristics and MBR in active thyroid eye disease group.

	Mild		Moderate to severe		Sight-threatening		*p* value
No. of eyes, *n* (%)	12		31		17		
Age, mean (SD)	42.6	(10.0)	46.8	(12.7)	57.3	(11.2)	0.003∗
Gender, male (%)	2	(16.7)	15	(48.4)	4	(23.5)	0.074
Smoking, *n* (%)	1	(0.1)	3	(0.1)	1	(0.1)	>0.999
History of high-dose intravenous steroid treatment, *n* (%)	3	(25.0)	15	(48.4)	12	(70.6)	0.052
History of orbital decompression, eyes (%)	0	(0)	1	(3.2)	5	(29.4)	0.014∗
Systemic findings							
SBP, mmHg, mean (SD)	98.5	(34.6)	140.4	(24.4)	122.3	(22.8)	0.002∗
DBP, mmHg, mean (SD)	86.2	(20.9)	82.3	(15.1)	74.0	(10.5)	0.503
MAP, mmHg, mean (SD)	85.5	(10.3)	100.7	(15.3)	90.1	(12.3)	0.119
MOPP, mmHg, mean (SD)	59.8	(34.2)	49.9	(10.5)	40.4	(8.7)	0.364
HR, bpm, mean (SD)	68.8	(14.6)	69.8	(9.2)	62.5	(11.6)	0.890
Ocular findings							
BCVA (logMAR), mean (SD)	0.08	(0.1)	0.08	(0.2)	0.29	(0.3)	0.005∗
IOP (primary gaze), mean (SD), mmHg	15.3	(3.8)	18.8	(4.9)	19.5	(3.4)	0.031∗
IOP (upward gaze), mean (SD), mmHg	19.4	(4.0)	22.7	(5.2)	25.7	(4.9)	0.033∗
MBR, mean (SD)							
MBR-overall	28.5	(2.7)	23.6	(3.2)	20.2	(4.3)	<0.001^†^
MBR-vessel	57.1	(8.0)	47.0	(6.9)	39.3	(6.9)	<0.001^†^
MBR-tissue	16.9	(3.1)	14.4	(2.9)	12.0	(2.7)	<0.001^†^

∗*p* < 0.05, ^†^*p* < 0.001. BCVA: best-corrected visual acuity; bpm: beats per minutes; DBP: diastolic blood pressure; HR: heart rate; logMAR: logarithm of the minimum angle of resolution; IOP: intraocular pressure; MAP: mean arterial pressure; MBR: mean blur rate; mmHg: millimeter of mercury; MOPP: mean ocular perfusion pressure: SBP: systolic blood pressure; SD: standard deviation.

## Data Availability

The data analyzed during this study are available on request from the corresponding author, Yi-Lin, Liao. The data are not publicly available due to it containing information that could compromise the privacy of research participants.

## Supplementary Materials

Supplementary 1 Supplemental Figure 1: mean blur rate (MBR) and different severities of active thyroid eye disease (TED). MBR-overall (a), MBR-vessel (b), and MBR-tissue (c) were significantly lower with more severe active TED. ^†^*p* < 0.001, ∗*p* < 0.05, ∗∗*p* = 0.058.

Supplementary 2 Supplemental Table 1: Spearman's rank correlation coefficient between clinical parameters and MBR.

Supplementary 3 Supplemental Table 2: Clinical characteristics and MBR in age-matched active thyroid eye disease group.
