# Supplementary figures and images for: Evaluation of Microcirculation in Optic Nerve Head Using Laser Speckle Flowgraphy in Active Thyroid Eye Disease
Source: Biomed Res Int. 2022 Mar 16;2022:9115270. doi: 10.1155/2022/9115270 (PMC8948602; doi:10.1155/2022/9115270)

**Supplemental Fig. 1**

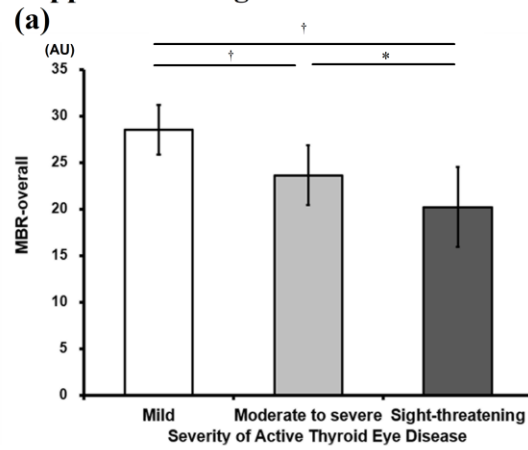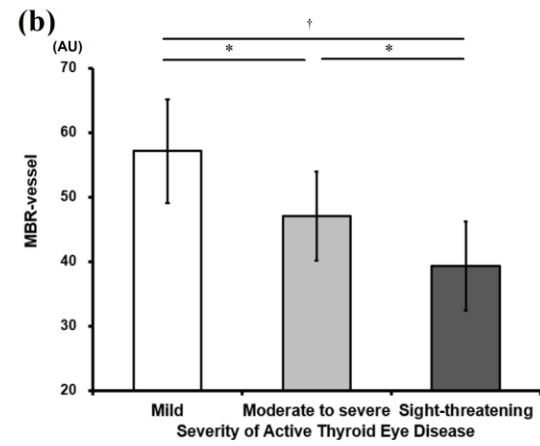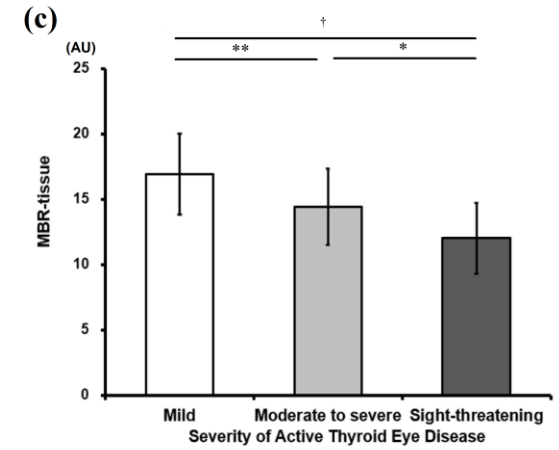

Supplement: Supplementary 1 — Supplemental Figure 1: mean blur rate (MBR) and different severities of active thyroid eye disease (TED). MBR-overall (a), MBR-vessel (b), and MBR-tissue (c) were significantly lower with more severe active TED. †p < 0.001, ∗p < 0.05, ∗∗p = 0.058. [file 9115270.f1.pdf]
