# Supplementary material for: Evaluation of Microcirculation in Optic Nerve Head Using Laser Speckle Flowgraphy in Active Thyroid Eye Disease
Source: Biomed Res Int. 2022 Mar 16;2022:9115270. doi: 10.1155/2022/9115270 (PMC8948602; doi:10.1155/2022/9115270)
Supplement: Supplementary 2 — Supplemental Table 1: Spearman's rank correlation coefficient between clinical parameters and MBR. [file 9115270.f2.pdf]

SUPPLEMENTAL TABLE 1. SPEARMAN'S RANK CORRELATION COEFFICIENT BETWEEN CLINICAL PARAMETERS AND MBR

MBR	Age	SBP	DBP	MAP	OPP	HR	BCVA (logMAR)	IOP (primary gaze)	IOP (upper gaze)
MBR-overall	-0.217	-0.253	-0.003	-0.321*	-0.180	0.195	-0.066	-0.193	-0.134
MBR-vessel	-0.342*	-0.212	0.105	-0.273	-0.201	0.137	-0.135	-0.121	-0.048
MBR-tissue	-0.168	-0.105	-0.114	-0.254	-0.134	0.139	-0.106	-0.222	-0.075

*$p < 0.05$

BCVA, best-corrected visual acuity; bpm, beats per minutes; DBP, diastolic blood pressure; HR, heart rate; logMAR, logarithm of the minimum angle of resolution; IOP, intraocular pressure; MAP, mean arterial pressure; MBR, mean blur rate; mmHg, millimeter of mercury; MOPP, mean ocular perfusion pressure; SBP, systolic blood pressure; SD, standard deviation
