# Supplementary material for: Evaluation of Microcirculation in Optic Nerve Head Using Laser Speckle Flowgraphy in Active Thyroid Eye Disease
Source: Biomed Res Int. 2022 Mar 16;2022:9115270. doi: 10.1155/2022/9115270 (PMC8948602; doi:10.1155/2022/9115270)
Supplement: Supplementary 3 — Supplemental Table 2: Clinical characteristics and MBR in age-matched active thyroid eye disease group. [file 9115270.f3.pdf]

SUPPLEMENTAL TABLE 2. CLINICAL CHARACTERISTICS AND MBR IN AGE-MATCED ACTIVE THYROID EYE DISEASE GROUP

	Mild	*Moderate to severe*	*Sight-threatening*	*p value*
No. of eyes, n (%)	7	7	7	
Age, years, mean (SD)	47.4 (8.0)	48.5 (8.0)	46.7 (8.9)	0.915
Gender, male (%)	2 (28.6)	3 (42.9)	4 (57.1)	0.854
Smoking, n (%)	1 (14.3)	4 (57.1)	1 (14.3)	0.260
History of high-dose intravenous steroid treatment, n (%)	2 (28.6)	4 (57.1)	5 (71.4)	0.285
History of orbital decompression, eyes (%)	0 (0)	0 (0)	1 (14.3)	>0.999
Systemic findings				
SBP, mmHg, mean (SD)	121.1 (19.2)	147 (32.9)	129.3 (13.3)	0.133
DBP, mmHg, mean (SD)	80.1 (8.4)	91 (18.6)	78.4 (12.8)	0.214
MAP, mmHg, mean (SD)	93.8 (11.6)	109.9 (22.1)	95.4 (12.4)	0.147
MOPP, mmHg, mean (SD)	45.9 (8.7)	53.3 (16.6)	43.9 (7.3)	0.308
HR, bpm, mean (SD)	69.0 (14.1)	66.9 (4.7)	63.0 (7.2)	0.503
Ocular findings				
BCVA (logMAR), mean (SD)	0.09 (0.16)	0.06 (0.07)	0.03 (0.04)	0.922
IOP (primary gaze), mean (SD), mmHg	16.0 (4.2)	19.8 (4.5)	20.6 (4.5)	0.137
IOP (upward gaze), mean (SD), mmHg	18.4 (4.8)	25.5 (5.2)	24.0 (6.4)	0.063
MBR, mean (SD)				
MBR-overall	27.7 (3.0)	22.9 (3.3)	20.1 (3.0)	0.001*
MBR-vessel	54.8 (9.4)	47.1 (7.2)	41.7 (7.9)	0.026*
MBR-tissue	16.3 (3.2)	13.2 (2.7)	12.3 (1.9)	0.030*

$*p < 0.05$

BCVA, best-corrected visual acuity; bpm, beats per minutes; DBP, diastolic blood pressure; HR, heart rate; logMAR, logarithm of the minimum angle of resolution; IOP, intraocular pressure; MAP, mean arterial pressure; MBR, mean blur rate; mmHg, millimeter of mercury; MOPP, Mean ocular perfusion pressure; SBP, systolic blood pressure; SD, standard deviation
